# Ring Chromosomes in Hematological Malignancies Are Associated with *TP53* Gene Mutations and Characteristic Copy Number Variants

**DOI:** 10.3390/cancers15225439

**Published:** 2023-11-16

**Authors:** Rachel J. Boyd, Jaclyn B. Murry, Laura A. Morsberger, Melanie Klausner, Suping Chen, Christopher D. Gocke, Andrew S. McCallion, Ying S. Zou

**Affiliations:** 1 McKusick-Nathans Department of Genetic Medicine, Johns Hopkins University School of Medicine, Baltimore, MD 21205, USA; rboyd25@jhmi.edu (R.J.B.); andy@jhmi.edu (A.S.M.); 2 Johns Hopkins Genomics, Baltimore, MD 21205, USA; jmurry3@jhu.edu (J.B.M.); lmorsber@jhmi.edu (L.A.M.); mhardy22@jhmi.edu (M.K.); schen123@jhmi.edu (S.C.); cgocke1@jhmi.edu (C.D.G.); 3 Department of Pathology, Johns Hopkins University School of Medicine, Baltimore, MD 21205, USA; 4 Cytogenetics Laboratory, Johns Hopkins Medicine, Baltimore, MD 21205, USA; 5 Department of Medicine, Johns Hopkins University School of Medicine, Baltimore, MD 21205, USA

**Keywords:** ring chromosomes, myeloid malignancies, gene mutation, complex karyotype, copy number variants

## Abstract

**Simple Summary:**

Ring chromosomes (RCs) are formed when chromosome ends fuse to form a circular structure. RCs are seen in <10% of patients with blood cancers (i.e., leukemias, lymphomas, and myelomas), and are associated with poor disease outcomes. We sought to evaluate how frequently RCs arise in patients diagnosed with these cancers and determine if there are any associated genetic variants, regions, or chromosomal abnormalities. Most patients with RCs have mutations in the *TP53* gene and gross chromosomal rearrangements, particularly among chromosomes 5, 7, 11, and 17. Most of these patients also possess marker chromosomes with genetic material of unknown origin. The majority of RCs that resemble identifiable chromosomes, as well as chromosomes lacking gross rearrangements, were seen in patients without *TP53* mutations. Our data suggest that mechanisms underlying RC generation may arise differently in patient groups with the presence or absence of functional *TP53*, a potentially important distinction for clinical decision making.

**Abstract:**

Ring chromosomes (RC) are present in <10% of patients with hematological malignancies and are associated with poor prognosis. Until now, only small cohorts of patients with hematological neoplasms and concomitant RCs have been cytogenetically characterized. Here, we performed a conventional chromosome analysis on metaphase spreads from >13,000 patients diagnosed with hematological malignancies at the Johns Hopkins University Hospital and identified 98 patients with RCs—90 with myeloid malignancies and 8 with lymphoid malignancies. We also performed a targeted Next-Generation Sequencing (NGS) assay, using a panel of 642 cancer genes, to identify whether these patients harbor relevant pathogenic variants. Cytogenetic analyses revealed that RCs and marker chromosomes of unknown origin are concurrently present in most patients by karyotyping, and 93% of patients with NGS data have complex karyotypes. A total of 72% of these individuals have pathogenic mutations in *TP53*, most of whom also possess cytogenetic abnormalities resulting in the loss of 17p, including the loss of *TP53*. All patients with a detected RC and without complex karyotypes also lack *TP53* mutations but have pathogenic mutations in *TET2.* Further, 70% of RCs that map to a known chromosome are detected in individuals without *TP53* mutations. Our data suggest that RCs in hematological malignancies may arise through different mechanisms, but ultimately promote widespread chromosomal instability.

## 1. Introduction

A ring chromosome (RC) is a cytogenetic abnormality that arises when double-stranded DNA breakpoints occurring on both arms of a linear chromosome have fused to form a circular structure. RCs can be classified as supernumerary rings (+r) or non-supernumerary rings (46, (r)). Supernumerary rings are predominantly composed of pericentromeric material that contributes to copy number (CN) gain [1], whereas most non-supernumerary rings (r) result in the loss of genetic material. In rare cases, non-supernumerary rings may exhibit telomeric or sub-telomeric fusion, resulting in the ability to function like the homologous chromosome due to the loss of very little genetic information [1]. 

All human chromosomes have shown evidence of RC formation in disease [1,2,3,4,5]. Constitutive RCs appear infrequently—with an incidence of approximately 1 in every 50,000 newborns—most of which are de novo [6,7]. Common phenotypic abnormalities associated with constitutive RCs include epilepsy, growth delays, intellectual disability, microcephaly, and craniofacial abnormalities [7]. RCs can derive from all human autosomes and sex chromosomes, and RCs have also been reported in cytogenetic profiles of human neoplasia. In fact, RCs arise so frequently in some mesenchymal tumors that the presence of an RC represents a cytogenetic hallmark that can aid in differential diagnoses [8,9]. 

In contrast, RCs are relatively rare events seen in <10% of hematological malignancies [10]. Despite this relatively infrequent occurrence, the appearance of an RC in patients with hematologic disorders is consistently associated with poor prognosis [8,11,12]. Because RCs are recurring rearrangements that may impact the prognosis of patients with hematological malignancies, gaining deeper insight into the cytogenetic and molecular characteristics of patients with such rearrangements is of interest. 

Meta-analyses suggest that RCs are present in approximately 7.4% of acute myeloid leukemias (AMLs), 6.8% of chronic lymphocytic leukemias (CLLs), 5.9% of myelodysplastic syndromes (MDSs), 5.1% of lymphomas, 3.4% of acute lymphoblastic leukemias (ALLs), and 1.9% of multiple myelomas (MMs) [8]. In cases with myeloid malignancies, 20.1% of observed RCs are estimated to be derived from chromosome 11, r(11), 14.9% from chromosome 7, 8.6% from chromosome 5, 7.8% from chromosome 21, and 6% from chromosome 18, whereas in lymphoid malignancies, 15% of RCs are estimated to be derived from chromosome 7, 12% from chromosome 1, 11% from chromosome 21, 8% from chromosome 9, and 5% from chromosome 8 [8]. 

RC formation has been thought to act as an alternative chromosome rescue mechanism for acrocentric chromosomes [1]; therefore, it is unsurprising that amplification of rRNA genes [13] clustered on the p arms of acrocentric chromosomes have been mapped to RCs in patients with a disease promoted by genomic instability. It is also unsurprising that amplification of proto-oncogenes, such as *KMT2A* (*MLL*) [14,15], a histone 3 lysine 4 methyltransferase with gene regulatory function during hematopoiesis on chromosome 11 and *RUNX1* (*AML1*)/*CBFA2* [16,17], a transcription factor involved in normal hematopoietic development on acrocentric chromosome 21, have been mapped to RCs in patients with these malignancies. To date, eight independent cases have also exhibited r(6) accompanied by t(15;17) [10]. Nevertheless, due to the scarcity of hematological malignancies with RCs, it remains challenging to elucidate consistent patterns in CN loss or gain, or genetic variants associated with RC formation in these disorders. 

Therefore, we sought to evaluate the incidence of RC formation in hematological malignancies diagnosed at the Johns Hopkins Hospital. We provide karyotype information for the largest cohort of patients with hematological malignancies and concomitant RCs to date. Because the molecular profiles of patients with RCs are not well characterized, we also sought to evaluate the suite of genetic variants and structural abnormalities carried by patients with a karyotype containing an RC, using Next-Generation Sequencing (NGS). Ultimately, this study provides a comprehensive profile of pathological gene mutations and copy number variants (CNVs) associated with hematological RCs.

## 2. Materials and Methods

### 2.1. Patient Cohort

Internally, we performed routine chromosome analysis from 13,124 hematological malignant specimens between 2014 and 2022. In total, 98 (0.75%) specimens, including 90 myeloid malignancies and 8 lymphoid malignancies, were selected for this study on the basis that they possessed RCs (Supplementary Table S1). Clinical, morphologic, immunophenotypic, cytogenetic, and/or molecular genetic features based on standard hematopathology practice, as well as the guidelines of the World Health Organization (the 4th edition), were used to classify these cases (Table 1). All procedures followed were in accordance with the ethical standards of the Institutional Committee on Human Experimentation and with the Helsinki Declaration of 1975. The research was approved by the Local Ethics Committee from the Johns Hopkins Hospital (IRB00398121), USA.

### 2.2. Cytogenetics Data

Bone marrow (BM) or peripheral blood (PB) specimens were collected from patients at diagnosis. Conventional chromosome analysis was performed on metaphase spreads from all patient samples, and karyotypes were described in strict accordance with the International System for Human Cytogenetic Nomenclature (version 2013, 2016, or 2020) (Supplementary Table S1) [18]. Interphase fluorescence in situ hybridization (FISH) and SNP microarray were performed, as described previously [19]. 

### 2.3. Next-Generation Sequencing 

DNA was extracted by conventional methods per manufacturer’s instructions (QIAcube; Qiagen, Hilden, Germany). DNA concentration was assessed using a Qubit fluorometer (Thermo Fisher Scientific, Waltham, MA, USA). NGS was performed on extracted genomic DNA, as outlined previously [19]. Briefly, library preparation was performed using Kapa Roche (Wilmington, MA, USA) reagents; hybrid capture was performed using Integrated DNA Technologies (IDT) probes (Coralville, IA, USA); libraries were sequenced using an Illumina NovaSeq (paired-end technology; Illumina, San Diego, CA, USA); and sequences were aligned to GRCh38/hg38. The targeted NGS assay used 40,670 IDT probes to cover a panel of 642 pan-cancer genes [19]. Mean read depth was 765× (range 341–1289), and 99.99% of target regions were captured at a level higher than 150×. Sequencing reads were visualized using the Integrative Genomics Viewer (IGV, Broad Institute, Cambridge, MA, USA). As previously described [20], oncogenic somatic variants were considered candidate somatic mutations if (1) variants were present with minimum variant allele frequency of ≥1%, in at least two alternate reads in both directions, and had an alternate allele base with mean Qscore of ≥11; (2) variants are described in COSMIC and/or ClinVar as being known cancer-associated mutations or mutational hotspots; and (3) variants were classified as deleterious and/or probably damaging by PolyPhen-2 [21] and/or SIFT [22] servers. 

### 2.4. Data and Statistical Analysis

All data were analyzed using R version 4.2.1 with custom functions and packages: ggplot2 version 3.4.0, stringr version 1.5.0, tidyr version 1.2.1, and dplyr version 1.0.10. The oncoplot was generated using a template from https://github.com/ptgrogan/excel-oncoplot, accessed on 20 October 2023. All data and analysis pipelines are available at https://github.com/rachelboyd/Hematological_Ring_Chromosomes, accessed on 12 November 2023. 

## 3. Results

### 3.1. Subtypes of RCs in Myeloid Malignancies with Various Chromosomal Abnormalities

The identified myeloid malignancies (*n* = 90) in decreasing frequency included AML (*n* = 47; 52.2%), MDS (*n* = 37; 41.1%), chronic myelomonocytic leukemia (CMML) converted to AML (*n* = 3; 3.3%), chronic myeloid leukemia (CML) (*n* = 1, 1.1%), and CML converted to AML (*n* = 2, 2.2%) (Table 1). Of the 90 patients with myeloid malignancies and RCs, 81 (90%) had complex karyotypes with greater than three independent cytogenetic abnormalities, 5 (5.6%) had 3 chromosomal abnormalities, and 4 (4.4%) had only 1 chromosomal abnormality in addition to the RC (Supplementary Table S1). NGS-based gene mutation results were available for 58 patients with RCs and myeloid malignancies. 

**Figure 1:**
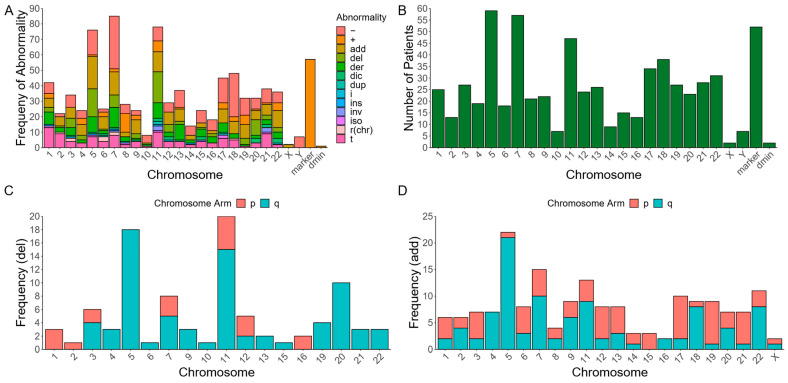
Chromosomal abnormalities in RC patients with myeloid-derived cancers. (**A**) Distribution of the frequency with specific chromosomal abnormalities is seen among 90 karyotypes from patients with myeloid-derived cancers and RC (dmin = double-minute chromosome; − = chromosomal deletion; + = chromosomal addition; add = addition of unknown origin; del = segmental deletion; der = derivative chromosome; dic = dicentric chromosome; dup = segmental duplication; i = isochromosome; idic = isodicentric chromosome; ins = insertion; inv = inversion; r (chr) = chromosome-derived RC; t = translocation). (**B**) Distribution of the number of myeloid-derived cancer patient karyotypes with at least one structural abnormality for a given chromosome. (**C**) The frequency of segmental deletions. (**D**) unknown additions seen in the p arms and q arms of affected chromosomes, across 90 karyotypes from patients with myeloid-derived cancers.

Common CNVs in these complex karyotypes included monosomy 7 (-7) or 7q deletion (7q-) (44.44%), -5/-5q (40.7%), -17/-17p (21%), as well as a gain of marker chromosome(s) of unknown origin (+mar) (63%), and structural abnormalities on chromosomes 11 (56.8%) and 18 (44.44%) (Figure 1A–D). Of these patients, 14 (17.3%) had multiple RCs. Roughly 13/14 (93%) individuals with multiple RCs had a +mar of unknown origin, 71% had a chromosome 22q abnormality, and 64% had a chromosome 11 abnormality. 

Two individuals in our cohort (case IDs 21 and 73) possessed double-minute chromosomes. Because double-minute chromosomes are frequently comprised of either *KMT2A*/*MLL* or *MYC* gene segments that are amplified in multiple copies, FISH was performed in these two cases using KMT2A and MYC probe sets. Both patients had normal CN for *KMT2A*, but amplification of *MYC*/*c-MYC* (Supplementary Figure S1), an oncogene that encodes a nuclear phosphoprotein involved in cell cycle progression, apoptosis, and cellular transformation.

Our data demonstrate that 89% of patients with myeloid malignancies and RCs in our cohort possessed ≥1 RC of unknown origin (+r). Of the 10 individuals who possessed rings that were able to be mapped to a cognate chromosome, 3 mapped to chromosome 7, 3 mapped to chromosome 6, 2 mapped to chromosome 3, 1 mapped to chromosome 2, and 1 mapped to chromosome 18 (Supplementary Table S1; Figure 1A). Interestingly, previous cases report hematological malignancies with +r(11), +r(7), +r(5), +r(18), and +r(21) [8]. However, in our dataset, the structural chromosomal abnormalities most frequently involve chromosomes 5, 7, 11, 18, and 17 (Figure 1B). 

In addition to chromosomal monosomies, segmental deletions and unknown additions are the most common structural abnormalities seen among these same chromosomes. Our data demonstrate that most add and del CNVs occur on specific arms of the chromosomes with the most chromosomal abnormalities, implicating a recurrent role for the involvement of cancer-associated loci (Figure 1C,D). The exception to this trend is add(17p) (Figure 1D), which involves the deletion of the chromosome 17p arm at the indicated location replaced by additional material of unknown origin (Figure 1D). These data suggest that RCs in myeloid malignancies are predominantly marker RCs associated with complex karyotypes and marker chromosomes with additional chromosomal instabilities among chromosomes 5, 7, 11, 17, and 18. 

### 3.2. Genetic Mutations among Patients with Myeloid Malignancies and RCs

**Figure 2:**
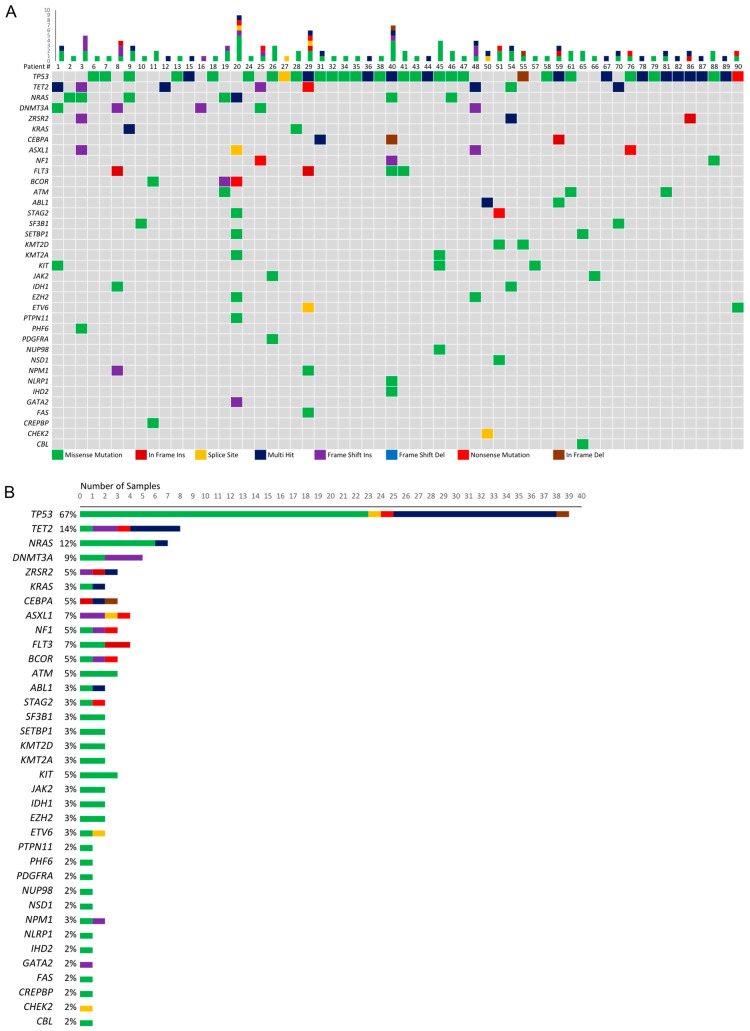
(**A**) Oncoplot of patients with myeloid-derived malignancies and RCs, including the (**B**) distribution of mutation type among each affected gene. Patient # was from the Table S1.

The majority of patients with myeloid malignancies and RCs with NGS data (*n* = 58) possess at least one candidate pathogenic mutation in *TP53* (*n* = 39 patients; 67.2%), which is normally involved in cell signaling in response to stress; *TET2* (*n* = 8 patients; 13.8%), which is normally involved in catalyzing the conversion of methylcytosine to 5-hydroxymethylcytosine; and/or *NRAS* (*n* = 7 patients; 12.1%), which normally functions as a GTPase (Supplementary Table S1). In fact, many of these patients exhibit multiple heterozygous or bi-allelic pathogenic mutations in *TP53*, *TET2*, and *NRAS* (Figure 2). Meanwhile, five or fewer patients possess putative pathogenic variants in additional genes that have been implicated in myeloid malignancies, such as *DNMT3A* (a DNA methyltransferase) [23], *KRAS* (a GTPase) [24], *CEBPA* (a transcription factor involved in cell cycle regulation) [25], *ASXL1* (a polycomb group protein involved in transcriptional regulation) [26], and *SF3B1* (a splicing factor involved in U2 snRNP formation) [27] (Figure 2). 

Four individuals lacked complex karyotypes, which showed the following: (1) 46,XY,−7,+r; (2) 46,XY,−18,+r; (3) 48,XX,+4,+r; and (4) 46,XX,r(18)(p11.3q23)[2]/46,XX,t(7;17) (q36;q21)[2]/46,XX[15]. Of these four patients, none possess a *TP53* mutation, whereas three (75%) have dissimilar pathogenic mutations in *TET2* and *DNMT3A* (two of which have two *TET2* mutations and one *DMNT3A* mutation), and the two patients without multiple *TET2* mutations share a g.209113113 G>A mutation in the NAD(+)-dependent isocitrate dehydrogenase, *IDH1,* that results in a p.R132C amino acid substitution (Supplementary Table S1).

**Figure 3:**
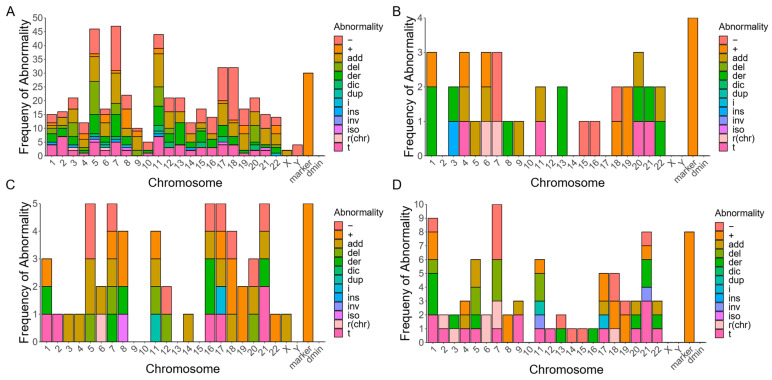
Gene mutations in RC patients with myeloid-derived cancers. (**A**) Distribution of the frequency of specific chromosomal abnormalities seen among 39 karyotypes from patients with myeloid-derived cancers that possess pathogenic variants in *TP53*. (**B**) Distribution of the frequency of specific chromosomal abnormalities seen among eight karyotypes from patients with myeloid-derived cancers that possess pathogenic variants in *TET2*. (**C**) Distribution of the frequency of specific chromosomal abnormalities seen among seven karyotypes from patients with myeloid-derived cancers that possess pathogenic variants in *NRAS*. (**D**) Distribution of the frequency of specific chromosomal abnormalities seen among 19 karyotypes from patients with myeloid-derived cancers that do not possess pathogenic variants in *TP53* (dmin = double-minute chromosomes; − = chromosomal deletion; + = chromosomal addition; add = addition of unknown origin; del = segmental deletion; der = derivative chromosome; dic = dicentric chromosome; dup = segmental duplication; i = isochromosome; idic = isodicentric chromosome; ins = insertion; inv = inversion; r (chr) = chromosomal-derived ring chromosome; t = translocation).

Among the 39 patients with *TP53* mutations, common structural abnormalities include monosomy 5, 7, 17, and 18, unknown additions to chromosomes 5, 7, 11, and 17, segmental deletions of chromosomes 5 and 11, and the acquisition of marker chromosomes (Figure 3A). Two patients in this group have RCs that map to a specific chromosome, r(3) and r(6). Aside from acquiring RCs and marker chromosomes, patients with *TET2* (Figure 3B) and/or *NRAS* (Figure 3C) mutations do not share common structural abnormalities; however, due to the small groups of patients with these characteristics, these results should be interpreted with caution. Included in these patient groups are three individuals with *NRAS* and *TP53* mutations, one with a *TET2* mutation and two *TP53* mutations, and one with a *TET2* mutation and an *NRAS* mutation.

Among the 19 patients that lack *TP53* mutations, marker chromosomes and monosomy 7 or partial chromosome 7 deletions are the most common structural abnormalities (Figure 3D). Interestingly, 70% of the chromosome-derived rings seen in this dataset are possessed by patients without *TP53* mutations. Specifically, these RCs map to chromosomes 2, 3, 6 (×2), 7 (×2), and 18 (Figure 3D). While this could arise from mechanistic differences between myeloid malignancies in patients with or without *TP53* mutations, it may also be the case that the chromosomal origin of an RC is more easily identified in non-complex karyotypes where there are fewer total chromosomal rearrangements and RCs are less likely to be the result of multi-segment rings. However, among these seven cases, four come from complex karyotypes and three come from non-complex karyotypes.

Perhaps unsurprisingly, these data suggest that the *TP53* locus and other known tumor suppressor genes and/or oncogenes are implicated in myeloid malignancies with RCs. Among all patients with NGS data, the presence of marker chromosomes and monosomy 7 are the most frequent cytogenetic abnormalities. These data also suggest that the mechanism of RC formation may be dependent on the presence of a *TP53* mutation—RCs that map to a specific chromosome are more likely to arise in patients without *TP53* mutations, whereas RCs of unknown origin are more likely to arise in patients with *TP53* mutations.

### 3.3. TP53 Mutations and Chromosome 17 Abnormalities among Patients with Myeloid Malignancies and RCs

*TP53* is a well-characterized tumor suppressor gene that plays critical regulatory roles in cell cycle regulation, DNA repair, and apoptosis. Mutations in *TP53* are the most common in human cancers (50–60%) [28]; however, disproportionately fewer patients with MDS and AML have mutations in *TP53* (<20%) [29,30,31,32]. In contrast, 67.2% of sequenced patients with myeloid malignancies and RCs in our dataset possess pathogenic variants in *TP53*, possibly due to the large proportion of patients with complex karyotypes. Among these patients, 64% possess one distinct *TP53* mutation and 36% possess two distinct *TP53* mutations; however, the two-hit hypothesis postulates that both *TP53* alleles must be lost to elicit a pathogenic effect, which is the case for >90% of human cancers. Because the chromosomal phase could not be determined from short-read NGS data, it is unclear whether the patients in our dataset are homozygous or compound heterozygous for their identified mutations. Therefore, we sought to look beyond whether patients possess a pathogenic *TP53* mutation and determine the frequency with which patients possess structural abnormalities of the 17p genomic region leading to *TP53* copy number loss (CNL).

**Figure 4:**
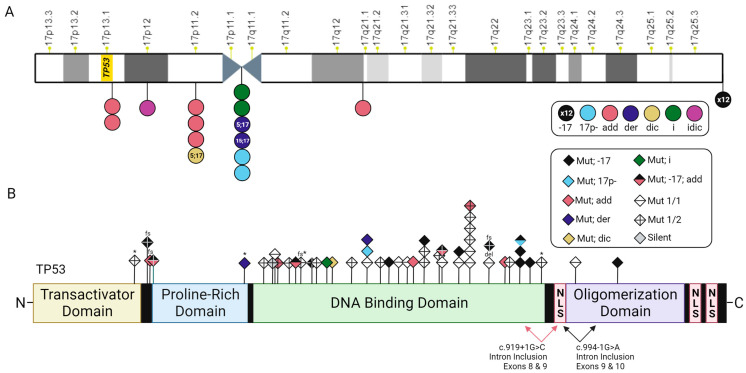
*TP53* mutations and chromosomal abnormalities in myeloid RCs. (**A**) Chromosome 17 ideogram indicating the cytogenetic position of *TP53* and various structural abnormalities (add = unknown addition; der = derivative chromosome; dic = dicentric chromosome; i = isochromosome; idic = isodicentric chromosome). (**B**) TP53 protein structure with the relative locations of amino acid changes associated with TP53 mutations (Mut = TP53 mutation; Mut 1/1 = the only TP53 mutation in that patient; Mut 1/2 = one of the two mutations in a patient; * = nonsense; del = deletion; fs = frameshift; NLS = nuclear localization signal).

We found that 21 patients with a *TP53* mutation (54%) also possess cytogenetic abnormalities indicative of *TP53* CNL (Supplementary Table S2). These include loss of the *TP53* locus at chromosome 17p13.1 through the formation of derivative, dicentric, and isochromosomes, as well as -17/-17p, and add (17) (Figure 4A, Tables S1 and S2).

**Figure 5:**
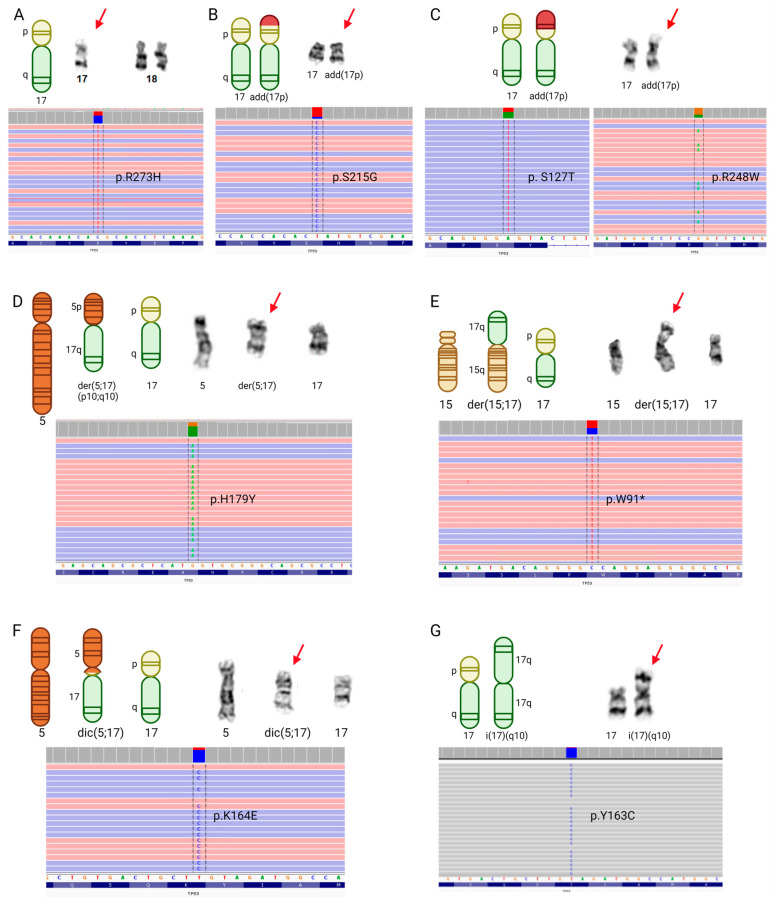
Structural rearrangements seen in karyotypes from myeloid RCs with TP53 mutations. (**A**) Monosomy 17 and a TP53 mutation in case 88; (**B**) An add(17p) and a TP53 mutation in case 38; (**C**) An add(17p) and TP53 mutation in case 36; (**D**) A derivative chromosome of 5 and 17 and a TP53 mutation in case 26; (**E**) A derivative chromosome of 15 and 17 and a TP53 mutation in case 90; * = nonsense mutation; (**F**) A dicentric chromosome of 5 and 17 and a TP53 mutation in case 24; (**G**) An isochromosome 17q and a TP53 mutation in case 76. Red arrows point to abnormal chromosomes, resulting in a loss of 17p, including TP53.

The majority of these patients’ karyotypes (*n* = 12; 57%) show monosomy 17 (Figure 5A), two of which also indicate add(17p) leading to loss of 17p13.1. An additional four patients have *TP53* CNL by add(17p) alone (19%; Figure 5B,C), two patients have a derivative chromosome 17 (9.5%; Figure 5D,E), and one patient has a dicentric chromosome 17 (4.8%; Figure 5F). Two patients have 17p-, and another two patients have isochromosome 17 (Figure 5G); however, only one of each pair of these individuals has a *TP53* mutation (Table S2). Among the patients with myeloid malignancies that lack NGS data, one individual has an isodicentric chromosome 17, leading to the loss of 17p (Figure 4A).

**Figure 6:**
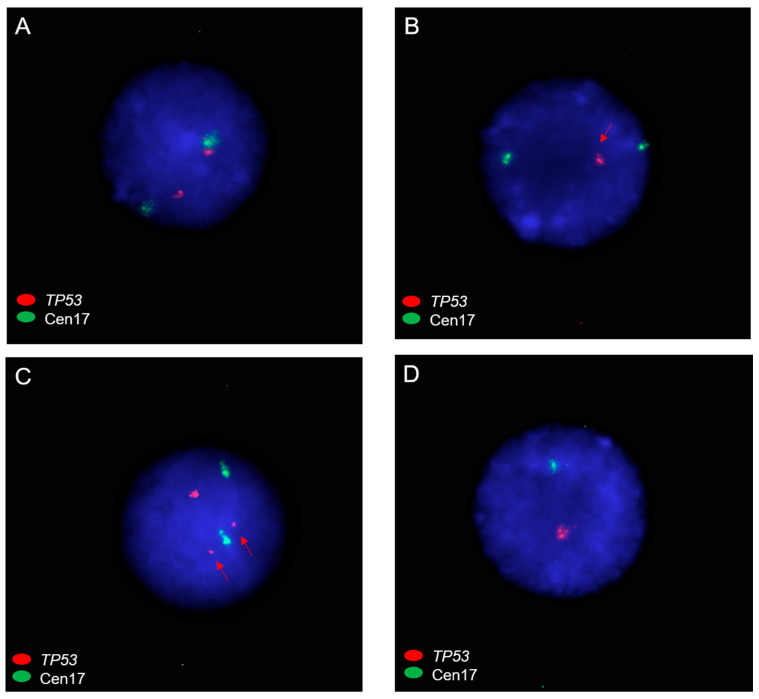
TP53 FISH in RC patients with add(17) where red = TP53 and green = centromere 17 (Cen17). (**A**) Normal copy number of TP53 (2R2G) with add(17q) (case 46). (**B**) Loss of TP53 (1R2G, red arrow points to TP53 signal) (case 27). (**C**) Abnormal TP53 copy number (2Rdim1R2G with red arrows pointing to two diminished fluorescence intensity signals of TP53) (case 38). (**D**) Loss of TP53 due to monosomy 17 (1R1G) (case 41). Cells were stained with 4′,6-diamidino-2-phenylindole (DAPI) in blue color.

For patients with add(17), we next employed FISH using TP53 and centromere 17 probes to further determine CNVs for TP53. A total of seven cases in our cohort had add(17p), and one case had add(17q). TP53 FISH was performed on available cell pellets of six add(17p) cases and one add(17q) case. The add(17q) case (case 46) had a normal TP53 CN (Figure 6A), as expected. Among cases with add(17p), three cases (case ID 27, 41, and 98) had CNL of TP53 with a FISH signal pattern consistent with the loss of 17p (Figure 6B), two cases (case ID 36 and 38) had an abnormal TP53 CN (with diminished fluorescence intensity of TP53) (Figure 6C), and one case (case ID 41) had CNL of TP53 with a FISH signal pattern consistent with monosomy 17 (Figure 6D). The two cases (case ID 36 and 38) with diminished fluorescence intensity signals of TP53 also had a normal fluorescence intensity of TP53, which may suggest complex abnormalities (such as rearrangements/CNVs) involving TP53 gene due to various other chromosomal abnormalities in RC cases by karyotype. Besides FISH, multiplex ligation-dependent probe amplification (MLPA), chromosomal microarray analysis (CMA), and whole-genome sequence data would be useful to further determine CNL status of TP53.

Next, we studied the mutational landscape of *TP53* in patients with myeloid-derived cancers and RCs to determine if mutations are enriched at particular residues or associated with specific cytogenetic abnormalities. A total of 54 *TP53* mutations exist among the 39 patients with mutations at this locus, 44 of which are unique (Supplementary Table S2). Overall, 38 of 54 mutations are missense (70%), 3 are nonsense, 3 cause a frameshift (1 of which leads to a premature stop), 2 result in intron inclusion, and 1 results in an in-frame deletion (Figure 4B). In concordance with previous reports that have characterized the mutational landscape of *TP53* in MDS [30] and AML [33,34], we found that the majority of TP53 mutations occur in residues within the DNA-binding domain (Figure 4B), with particular enrichment for g.7577538 C>T/g.7577539 G>A/C (p.R248W/Q/G; *n* = 6), g.7578190 T>C (p.Y220C; *n* = 3), and g.7577120 C>T/A (p.R273H/L; *n* = 3). All three patients with missense mutations at g.7577120 (p.R273) have corresponding monosomy for chromosome 17 (Figure 4B; Supplementary Table S2). Otherwise, there appears not to be a relationship between cytogenetic abnormalities and particular *TP53* mutations.

Taken together, these data suggest that patients with myeloid malignancies may acquire RCs of unknown origin through cell cycle instability resulting from loss of function (LOF) *TP53* mutations combined with CNL of *TP53* at 17p13.1. While there does not appear to be one specific cytogenetic abnormality coupled with RCs, mutations in TP53 that affect residues in the DNA-binding domain, or cause premature termination or frameshifts, are associated with chromosomal instability that may promote RC formation.

### 3.4. Subtypes of RCs in Lymphoid Malignancies with Various Chromosomal Abnormalities

The identified lymphoid malignancies (*n* = 8) in decreasing frequency included MMs (*n* = 5; 62.5%), ALLs (*n* = 2; 25.0%), and CLLs (*n* = 1; 12.5%). Among the eight patients with lymphoid-derived malignancies, 100% had complex karyotypes with exactly one ring of unknown origin (+r), and only two (25%) patients had an associated +mar (Supplementary Table S1; Supplementary Figure S2A). The majority of patients possessed at least one structural abnormality on chromosome 8 (87.5%), chromosome 6 (75%), and chromosome 22 (62.5%) (Supplementary Figure S2B). Common aneuploidies in these patients include monosomy 13 or 13q deletion (13q-) (75%), nullisomy/monosomy 22 (50%), and monosomy 4 (37.5%) (Supplementary Figure S2A). Other common structural abnormalities include add(1) and add(8) (Supplementary Figure S2A). In contrast with the data from patients with myeloid-derived malignancies, among the patients with lymphoid-derived malignancies, segmental deletions (Supplementary Figure S2C) and additions of unknown origin (Supplementary Figure S2D) were primarily enriched in the p arms of the affected chromosomes. NGS data were only available for one patient with a singular *TP53* mutation affecting the resulting protein’s DNA binding region (g.7577095 G>C; p.D281E). Although a larger sample size of patients with lymphoid malignancies and RCs would permit more thorough analyses and confidence in the observed trends, these data suggest that RC formation in hematological disorders may arise through different mechanisms between patients with lymphoid- and myeloid-derived malignancies.

## 4. Discussion

Our study provides karyotype data and corresponding mutational profiles for the largest cohort of patients with hematological malignancies and concomitant RCs to date. Additionally, our data provide further biological insight into the presence of RCs in this patient group. Through a combination of karyotype analysis and detection of putative pathogenic variants from NGS data, we have begun to identify genetic mutations and chromosomal abnormalities that may contribute to the pathogenicity of RCs in hematological malignancies.

Our data reveal that most patients with RCs possess complex karyotypes that indicate significant chromosomal abnormalities, including marker chromosomes; complex structural arrangements of chromosomes 5, 7, and 11; and CNVs among chromosomes 5, 7, 11, 17, and 18. Previous reports have similarly found a high frequency of cytogenetic abnormalities associated with chromosomes 5, 7, 11, and 17 in AML and MDS patient karyotypes [14,17,19,35,36]. Among previous studies that have specifically characterized AML and MDS patients with RCs, one similarly found an association between RCs and complex karyotypes, observed that 79% of their patient cohort had RCs of unknown origin [37] (compared to 89% of our cohort), and saw that 73% of their cohort possessed concomitant marker chromosomes (compared to 63% of our cohort), while the other found that most RCs in their patient cohort mapped to chromosomes 5, 7, 11, 18, and 21 [8]. An increasing number of studies have also shown that r(6) is often accompanied by t(15;17) [10]; however, no patient in our study possessed r(6) accompanied by t(15;17).

Our data further demonstrate that among add and del CNVs on chromosomes 5, 7, 11, and 17, most of these events occur at the q arm of affected chromosomes, with 17p being a notable exception. This is of particular significance, because the *TP53* tumor suppressor gene is found at 17p13.1. In fact, an analysis of NGS data reveals that the majority of patients in our cohort have putative pathogenic mutations in *TP53*. Most *TP53* mutations are missense mutations that affect amino acid residues within the DNA-binding domain of the resulting protein, or frameshift and nonsense mutations that lead to TP53 LOF. Notably, there is evidence that a similar proportion of AML patients with complex karyotypes and poor prognoses tend to possess *TP53* alterations (~70%) [38], and these patients also possess similar CN alterations, including -5/5q-, -7/7q-, -18/18q-, and +11/+11q/amp11q13∼25. Many of the pathogenic mutations identified in our dataset have been seen in other AML and MDS patient cohorts [29,30,31,32,33,34]; however, our study is the first to specifically characterize the distribution of *TP53* mutations in patients with RCs.

We also demonstrate that by integrating mutation data and karyotype data, we were able to provide evidence of 24 patients, 3 of whom do not possess NGS data or a *TP53* mutation, with chromosomal aberrations leading to the loss of one or both copies of *TP53*. In many of these patients, 17p CNVs and/or monosomy 17 may represent the “second hit” required for the pathogenic effect of the “first hit” that is a *TP53* mutation. Conversely, a subset of patients lacking *TP53* mutations possess mutations in other known genes implicated in myeloid malignancies, including *TET2*, *NRAS*, and *DNMT3A* [20,29,32]. Interestingly, we observe that the patients without *TP53* mutations or 17p CNL structural abnormalities are more likely to possess chromosome-specific RCs and non-complex karyotypes.

Taken together, our data suggest that multiple mechanistic subtypes of RCs may exist in hematological malignancies. While there do not appear to be chromosomal hotspots associated with RCs in myeloid- or lymphoid-derived malignancies at large, or among patients with and without *TP53* mutations (Supplementary Figure S3), the mechanism of RC formation, and the role RCs play in these diseases may differ. RCs are known to arise through a variety of catastrophic genome cohesion/repair failures (e.g., chromoanagenesis). For example, there is evidence suggesting that marker chromosomes and ring chromosomes can both arise from chromothripsis, that *TP53* mutations promote chromothripsis events, and that all of these events are independently associated with poor prognosis in myeloid malignancies [39,40]. Furthermore, there is evidence that marker chromosomes can form ring structures [41,42,43]. Therefore, we postulate that in individuals with *TP53* mutations and/or chromosome 17 CNL, the accumulation of these events may lead to cell cycle instability, resulting in chromothripsis and the subsequent formation of marker chromosomes that form derivative ring structures upon subsequent cell divisions.

Moreover, in patients without a *TP53* mutation, cell cycle instability may arise through pathogenic mutations in other tumor suppressor genes or may be promoted by oncogene amplification mapped to a chromosome-derived RC, which may explain the segregation of RC mapping to cognate chromosomes in these patients. In fact, it is well known that cell cycle checkpoint deficiencies and short telomeres may lead to chromosome end-joining and the formation of RCs, chromosome bridges, isochromosomes, polyploidy, dicentric chromosomes, and other structural rearrangements in many cancers [7,9,44]. For example, TET2 is another tumor suppressor gene, mutations in which are thought to lead to hematopoietic stem cell proliferation deficiencies [45] and may prevent genomic stability by impeding the addition of 5-hydroxymethylcytosine at sites of DNA damage in cancer cells [46]. Constitutive RC in disease is known to be associated with growth delays and increased cell death [47,48]; therefore, it is unsurprising that mutations in tumor suppressor genes and genes involved in cell cycle checkpoints may allow RCs to persist through cell cycle checkpoints in hematological malignancies.

RC formation and pathogenicity in lymphoid malignancies may arise through different mechanisms from those with myeloid malignancies; however, a larger sample size of patients with lymphoid malignancies and RCs would be required to observe real differences between these groups. In addition to acquiring larger cohorts of patients with lymphoid malignancies, high-throughput genomic strategies could be employed to compare the molecular profiles of patients with myeloid vs. lymphoid malignancies and RCs. Ultimately, by assessing the landscape of patients with hematological malignancies and RCs, we propose that RC formation may be a cause and/or effect of cell cycle instability in these patients, with the direction of this relationship determined by the presence of *TP53* mutations. Finally, due to the scarcity of RCs in hematological malignancies, our dataset represents a resource that will be particularly valuable to future analyses that aim to assess the cytogenetic and mutational landscapes of patients with these abnormalities. Future studies that accumulate more RCs from multi-centers, obtain comprehensive clinical data, and follow various treatment strategies in patients with RCs will shed light on treatment response rates, survival rates, and overall prognosis of these patients.

## 5. Conclusions

A subset of patients with hematological malignancies and RCs (0.75%) was identified in our institution. A total of 92% of these patients had myeloid-derived cancers, associated with complex karyotypes and recurrent CNVs involving chromosomes 5, 7, 11, 17, and 18. Among patients with myeloid-derived malignancies for which NGS data were available, 93% had complex karyotypes and 72% of these individuals had pathogenic mutations in TP53. By integrating NGS and karyotype data, we found that 54% of individuals with TP53 mutations also possessed cytogenetic abnormalities leading to TP53 CNL. All patients with RCs that lacked complex karyotypes also lacked TP53 mutations but had pathogenic mutations in TET2, and of the 10 RCs that were able to be mapped to a cognate chromosome, 70% were found in karyotypes from patients without TP53 mutations. Most individuals in our dataset (63%) had at least one +mar, including 77% of individuals with TP53 mutations and 93% of patients with multiple RCs of unknown origin, suggesting that TP53 deficiency may promote a cascade of genomic instability that includes marker chromosome and RC formation.

Overall, this study identified specific molecular profiles that may be associated with distinct RC subtypes in myeloid malignancies. We postulate that these subtypes may be defined by TP53 mutational status and the genomic composition of RCs. These data indicate that future studies of the different myeloid RC subtypes’ molecular profiles and their impact on disease outcomes are needed to inform clinical decision making. Future studies should evaluate the presence of cryptic imbalances and genomic content involved in those affected with hematological malignancies and RCs.

## Figures and Tables

**Table 1:** WHO classification of 98 patients with ring chromosomes in this study.

WHO Classification	Total Patients (%)	Mean Age at Diagnosis (Age Ranges)
Myeloid malignancies		
Acute myeloid leukemia (AML)	47 (48.0)	67 (44–88)
Myelodysplastic syndromes (MDS)	37 (37.8)	70 (34–86)
Chronic myelomonocytic leukemia (CMML) → AML	3 (3.1)	62 (42–76)
Chronic myeloid leukemia (CML) → AML	2 (2.0)	60 (56–66)
CML	1 (1.0)	61
Lymphoid malignancies		
Multiple myeloma (MM)	5 (5.1)	61 (42–74)
Acute lymphoblastic leukemia (ALL)	2 (2.0)	40 (6–74)
Chronic lymphocytic leukemia (CLL)	1 (1.0)	70

## Data Availability

The dataset for the current study is available at https://github.com/rachelboyd/Hematological_Ring_Chromosomes, accessed on 12 November 2023.

## Supplementary Materials

The following supporting information can be downloaded at: https://www.mdpi.com/article/10.3390/cancers15225439/s1, Supplementary Table S1: Karyotype information and corresponding NGS data for 98 patients with hematological malignancies; Supplementary Figure S1: Double-minute chromosomes in RC patients; Supplementary Figure S2: Distribution of chromosomal abnormalities seen among eight karyotypes from patients with lymphoid-derived cancers and RCs; Supplementary Figure S3: Chromosome size-normalized distribution of the frequency that specific chromosomal abnormalities are seen among patients with myeloid- and lymphoid-derived cancers and RCs; Supplementary Table S2: TP53 mutations and co-occurring structural abnormalities on chromosome 17.
