# Supplementary material for: Ring Chromosomes in Hematological Malignancies Are Associated with TP53 Gene Mutations and Characteristic Copy Number Variants
Source: Cancers (Basel). 2023 Nov 16;15(22):5439. doi: 10.3390/cancers15225439 (PMC10670249; doi:10.3390/cancers15225439)
Supplement: Supplementary file 1 [file cancers-15-05439-s001.zip › cancers-2676276-supplementary.pdf]

Table S1. Karyotype information and corresponding NGS data for 98 patients with hematological malignancies.

Case ID	Karyotype	Neoplasia	Mutations Identified by NGS
	**Myeloid-Derived**		
1	48,XX,+4,+r[20]	AML	KIT g.55599320 G>T (p.D816Y)<br>TET2 g.106158226 CATAAGGCTCT>C (p.H1043fs)<br>TET2 g.106158456 AT>A (p.L1120fs)<br>DNMT3A g.25467436 A>C (p.L547R)
2	49~51,XY,+18,+3~5mar[14]/47,XY,+r[6]	AML	NRAS g.115258747 (p.G12A)
3	48~51,XY,+18,+19,+1~2r,+1~2mar[cp20]	AML	NRAS g.115258744 C>T (p.G13D)<br>PHF6 g.133551238 T>C (p.C292R)<br>ASXL1g.31022441 A>AG (p.G646fs)<br>TET2 g.106180783 T>TG (p.C1271fs)<br>ZRSR2 g.15840894 C>CA (p.N327fs)
4	48,XX,+4,+r[4]/48,sl,del(11)(p14p11.2)[16]	AML	
5	46,XX,?inv(11)(q23q24),-18,?inv(21)(q21q22),+r[20]	AML	
6	44,XX,-5,-7,del(11)(q14q23),add(18)(q21.1),-20,+r[19]	AML	TP53 g.7578394 T>C (p.H179R), -17p^
7	43~49,XY,del(5)(q31q33),-7,-13,-18,+1~2r,+1~5mar[cp18]	AML	TP53 g.7578368 (p.H179R)
8	46,XX,t(7;17)(q36;q21)[2]/46,XX,r(18)(p11.3q23)[2]/46,XX[15]	AML	FLT3 351bp amplicon (24 bp ITD)<br>IDH1 g.209113113 G>A (p.R132C)<br>NPM1 g.170837543 C>CTCTG (p.W288fs)<br>DNMT3A g.25468172 C>CA (p.V502fs)
9	46,XY,add(4)(q25),-5,+11,-12,?t(17;21)(q25,q11.2),+mar[9]/47,sl,+r[11]	AML	NRAS g.115258747 (p.G12D)<br>KRAS g.25380275 (p.Q61H)<br>KRAS g.25398261 (p.T20A)<br>KRAS g.25398285 (p.G12C)<br>TP53 g.7577539 G>A (p.R248W)
10	46,XY,del(7)(p11.2)[9]/46,XY,r(7)(p11.2q36)[4]/46,XY,-7,+mar[6]/46,XY[1]	AML	SF3B1 g.198266486 T>C (p.M784V)
11	44~45,XY,add(5)(q?22),-7,-13,-14,?add(17)(p11.2),+r,+1~2mar[16]/46,XY[4]	AML	BCOR g. 39932564 C>T (p.V679I)<br>CREBBP g.3900384 C>G (p.V238L)
12	46~49,X,-X,+1~2r,+1~2mar[cp18]/48,XX,der(21)?t(20;21)(q12;q22),+r,+mar[2]	AML	TET2 g.106157113 CA>C (p.H672fs)<br>TET2 g.106182972 T>G (p.Y1337*)
13	45,XY,del(3)(p12),psu dic(7;11)(p22;q24)ins(7;?)(p22;?),-18,+r,+mar[19]/46,XY[1]	AML	TP53 g.7578236 A>C (p.Y205D)
14	46~47,XX,-5,del(5)(q15q33),+8,add(9)(q34),del(12)(q24.1),add(19)(p13.3),+r[cp20]	AML	
15	46,XY,del(20)(q11.2q13.2)[6]/46,sl,del(11)(q13q23),add(21)(p11.2),+r[2]/46,XY[12]	AML	TP53 g.7577538 C>T (p.R248Q)<br>TP53 g.7578449 C>T (p.A161T)
16	46,XX,der(11)?inv(11)(q23q24),?der(21)add(21)(q22)[13]/46,sl,-18,+r[6]/46,XX[1]	AML	DNMT3A g.25467519 GT>G (p.N519fs)
17	46,XX,inv(11)(q23q24),?inv(21)(q21q22)[13]/46,sl,-18,+r[5]/46,sl,t(1;2)(p13;p13)[2]	AML	
18	44~46,XY,-3,der(5)t(3;5)(q11.2;q13),+8,add(9)(q12),-17,-18,+mar,+r[cp19]/46,XY[1]	AML	TP53 g.7577120 C>A (p.R273L)<br>-17p^
19	46,XY,del(5)(q22q35),del(7)(q22q36)[4]/46,sl,-del(5),dup(11)(q23q24),+r[15]/45,XY[1]	AML	NRAS g.115258747 C>G (p.G12A)<br>BCOR g.39934232 G>GC (p.Q123fs)<br>ATM g.108170474 C>T (p.P1680L)
20	47,XY,+8[5]/47,sl,+1,der(1;16)(q10;p10),idic(17)(p12)[9]/48,sdl,?r(6),-idic(17),+17,+19[6]	AML	BCOR g.39923059 G>A (p.R1183*)

			NRAS g.115258747 C>T (p.G12D)<br>NRAS g.115258748 C>T (p.G12S)<br>PTPN11 g.112888163 G>T (p.G60V)<br>SETBP1 g.42531907 G>A (p.D868N)<br>STAG2 g.123217380 C>T (p.R1012*)<br>ASXL1 g.31022233 A>C (c.1720-2A>C)<br>GATA2 g.128204735 T>TA (p.M236fs)<br>KMT2A g.118343943 T>C (p.M690T)<br>EZH2 g.148512040 A>T (p.N546K)
21	48,XX,+19,+r[7]/47,XX,+19,6~13dmin[5]/49~50,XX,+mar1x3~4[6]/48,XX,+mar1,+mar2[2]	AML	
22	44,XY,dic(3;13)(q29;p11.2),del(5)(q15q31),der(7;11)(p10;q10)[12]/46~47,sl,-11,+1~2r,+2mar[8]	AML	
23	43~45,XX,der(5;17)(p10;q10),add(6)(p21),add(7)(q11.1),-11,-19,-22,-22,+r,+1~2mar[cp5]/46,XX[15]	AML	
24	42~44,X,-Y,-4,dic(5;17)(q11.2;p11.2),+8,add(9)(p24),-11,der(12;22)(q10;q10),-15,+r,+1~2mar[cp20]	AML	TP53 g.7578440 T>C (p.K164E)
25	45,XX,-7[10]/45,sl,add(5)(q34),add(9)(q22),add(20)(q13.2)[7]/45,sl,add(4)(q21),t(4;11)(q21;q23),r(6),-15,<br>+mar[3]	AML	NF1 g.29665757 C>A (p.Y2285*)<br>DNMT3A g.25457242 C>T (p.R882H)<br>TET2 g.106194045 GA>G (p.E1504fs)
26	43~44,XX,der(5;17)(p10;q10),-6,add(6)(p?22),-7,add(7)(q11.1),-11,-19,-22,-22,?add(22)(q11.2),+r,<br>+2~3mar[cp20]	AML	JAK2 g.5073770 G>T (p.V617F)<br>TP53 g.7578395 G>A (p.H179Y)<br>PDGFRA g.55156534 C>T (p.R979C)
27	41,XY,add(3)(q21),del(5)(q22q35),-7,-11,dic(12;15)(p11.2;p11.2),-16,add(17)(p11.2),-18,-20,+r[12]/40,sl,-Y[3]/<br>46, XY[5]	AML	TP53 g.7577018 C>G (c.919+1G>C)
28	43~46,XX,del(3)(q21q26.2),del(4)(q31),del(5)(q13q31),-7,-8,dup(11)(q13q21),-17,-18,add(21)(p11.2),+r,<br>+1~2mar [cp20]	AML	KRAS g.25398280 (p.G13D)<br>TP53 g.7578190 T>C (p.Y220C)
29	43~48,XX,der(3)ins(3;?)(p13:?),+6,add(6)(p21.3)x2,der(8;13)(q10;q10),add(11)(q24),-16,+19,add(22)(q11.2),<br>+1~2r [cp20]	AML	TP53 g.7578553 T>C (p.Y126C)<br>ETV6 g.12006497 T>C (c.463+2T>C)<br>TET2 g.106158025 C>T (p.Q976*)<br>TP53 g.7578427 T>C (p.H168R)<br>FAS g.90774066 A>C (p.E289D)<br>FLT3 351bp amplicon (24 bp ITD)<br>NPM1 fs
30	47,XY,t(1;6)(p36.1;q25),add(9)(p21),+11,del(16)(p13.1)[10]/46,XY,t(1;21)(p32;q22),add(5)(q12),7,<br>del(19)(q13.2),+r[5]/46,XY[5]	AML	
31	45,XY,add(2)(q23),-5,der(7)t(7;11)(q11.2;q13),add(13)(p11.2),add(18)(q23),del(20)(q11.2q13.3),21,+r[12]/<br>45,idem,del(9)(q12q31)[4]	AML	CEBPA g.33792308 A>G (p.L338P)<br>TP53 g.7577539 G>A (p.R248W)<br>CEBPA g.33792437 G>A (p.A295V)
32	45,XX,add(2)(p23),del(5)(q13q33),del(7)(q22),-18,der(19)t(18;19)(q11.2;q13.4)[8]/45,sl,add(14)(q32),17,+r[6]/<br>46, sdl1,+mar[4]/46,XX[2]	AML	TP53 g.7578271 T>A (p.H193L)
33	38~39,XX,add(2)(p23),-3,add(4)(q27),del(5)(q13q31),-7,-8,-8,-10,-11,add(12)(p11.2),-13,-16,-17,-18,-19,-20,22,<br>+r,+4~5mar[16]/46,XX[4]	AML	
34	45~46,XX,-4,der(5)t(4;5)(q12;q13),add(7)(p15),der(10)t(10;10)(p13;q11.2),-11,add(11)(q14),add(13)(q21),-18,<br>+r,+1~2mar[cp15]/46,XX[5]	AML	TP53 g.7577548 C>T (p.G245S)
35	45,XY,-2,-3,del(5)(q13),der(6)t(2;6)(p15;p23),der(7)add(7)(p11.2)add(7)(q22),-12,-14,<br>der(16)t(14;16)(q24;q24),+22, der(?)t(?;12)(?;q11),+r[20]	AML	TP53 g.7574027 C>G (p.G334R)
36	45,XY,der(5)add(5)(q21)t(5;18)(p15;q12),-8,dic(12;22)(p11.2;p11.2),-18,-21,+r,+2mar[5]/45,sl,<br>add(17)(p11.2)[13]/45,sl,-add(17)(p11.2),+add(17)(p13)[2]	AML	TP53 g.7578551 A>T (p.S127T)<br>TP53 g.7577539 G>A (p.R248W)
37	43~46,XY,t(3;18)(q21;q23),-4,?del(4)(q33),add(5)(q13),der(7;17)(p10;q10),dic(9;12)(q13;p11.2),del(10)(q22),<br>?del(12)(p13),-13,del(13)(q12q14),+r,+1~2mar[cp20]	AML	

38	41~45,XX,del(1)(p32p36.1),add(5)(q13),-6,+8,-9,del(12)(q15q24.1),-13,der(15)t(13;15)(q12;q24), t(15;18) (q24;q11.2),add(17)(p13),-18,add(19)(q13.3),+r[cp19]/46,XX[1]	AML	TP53 g.7578201 (p.S215G)
39	42~46,XY,add(5)(q11.2),add(5)(q22),+7,-9,del(11)(q13q23),-13,add(18)(q12),+20,del(20)(q11.2q13.2)x2,-21, -21,dic(21;22)(p11.2;p11.2)del(21)(q22),add(22)(p11.2),+r[cp18]/46,XY[2]	AML	
40	44~45,XX,add(X)(p22.1),t(1;2)(q25;q31),add(5)(q13),-7,add(11)(p15),add(14)(p11.2),-16,-17,-18, add(18)(q23),-20, add(20)(p13),add(21)(p11.2),+22,psu dic(?;18)(?;p11.3),+r,+mar[cp20]	AML	FLT3 g.28592642 C>A (p.D835Y)<br>IDH2 g. 90631934 C>T (p.R140Q)<br>NRAS g.115258748 C>T (p.G12S)<br>NF1 g.29587531 AGGT>TCCGA (p.R1505fs)<br>TP53 g.7574034 C>T (c.994-1G>A)<br>TP53g.7579513 TG>T (p.P58fs)<br>CEBPA g.33792754 GGGCGGCGGC>G (p.P187_P189del)<br>NLRP1 g.5462640 C>T (p.R459Q)
41	43~46,XX,add(3)(p21),-4,add(5)(q13),-7,del(11)(p11.2p15),add(12)(p13),der(13;22)(q10;q10), ?add(16)(q11.1),-17, add(17)(p11.2),-18,-19,add(20)(p11.2),i(21)(q10),+1~2r,+2~5mar[cp20]	AML	FLT3 g.28626716 C>T (p.V194M)<br>TP53 g.7578496 A>G (p.L145P)
42	44~46,X,-Y,add(2)(q23),add(4)(q21),-5,del(6)(q13),der(7)t(7;11)(q11.2;q13),del(9)(q12q31),add(13)(p11.2), der(13)add(13)(p11.2)add(13)(q34),add(18)(q23),del(20)(q11.2q13.3),-21,+mar,+r[cp20]	AML	
43	46,XY,der(1;13)(q10;q10),add(5)(q11.2),-7,-12,-18,+4mar[6]/45,XY,add(3)(p21.3),add(5)(q31.1),add(7)(q22), -8,-12, add(12)(p11.2),der(13)t(7;13)(q22;q34),-15,-16,-17,-19,-20,+r,+5mar[7]/46,XY[7]	AML	TP53 g.7577548 (p.G245S)
44	42~49,add(X)(q13),Y,del(2)(p23),+4,del(5)(q15q33),+add(6)(q27),dup(6)(p22p23)x2,-7,+8,+9,add(11)(p15), -13,-15, -17,add(17)(p13),add(20)(q13.1),del(20)(q11.2),del(22)(q13.1q13.3),+1~2r, +1~2mar[cp20]	AML	TP53 g.7577568 C>T (p.C238Y)<br>TP53 g.7578271 T>C (p.H193R)
45	43-47,XY,der(3)r(3;?)(p26q29;?),add(6)(q23),der(6)t(6;7)(p25;q11.1),add(7)(q22),add(9)(q34),-10, del(11)(p11.1),dic(11;15)(p11.2;p11.2),-13,-14,-17,-19,add(19)(p13.1),add(19)(p13.3),add(21)(p11.2), +1~3mar,inc[cp12]	AML	NUP98 g.3716782 C>T (p.R1355Q)<br>TP53 g.7577120 C>T (p.R273H)<br>KIT g.55594177 C>G (p.P627R)<br>KMT2A g.118374578 G>C (p.K2657N)
46	44-47,XY,add(3)(q21),-5,add(5)(q31),add(6)(p21),+7,add(7)(q22),der(7;8)(p10;q10)x2,+8,i(8)(q10)x2, del(11) (q23q?25),del(12)(p11.2p12),add(16)(q24),der(16)t(16;21)(q12;q11.2),add(17)(q21),?19, del(20)(q11.2),-21,+r, +1~3mar[cp20]	AML	NRAS g.115258744 (p.G13D)<br>TP53 g.7577129 (p.F270C)
47	41,X,-Y,del(5)(q13q33),add(7)(p11.2),add(8)(p11.2),add(13)(p11.1),-14,-15,-17,-18,-19,add(20)(q13.2),der(20) t(17;20)(q21;q13.1),-21,+ider(?)(p?)t(?;15)(?;q11.2),+mar[11]/40,sl,dic(8:20)(q24.3;p13)add(8)(p11.2)t(17;20) (q21;q31.1)[6]/41,sdl,+r[3]	AML	TP53 g.7573996 (p.L344P)
48	46,XY,-7,+r[20]	CMML→AML	ASXL1 g.31022441 A>AG (p.G646fs)<br>DNMT3A g.25467033 GTCGTGG>GGTGTTGT (p.N612fs)<br>TET2 g.106196726 C>T (p.Q1687*)<br>TET2 g.106190860 C>T (p.H1380Y)<br>EZH2 g.148507466 T>G (p.Y663S)
49	46,XY,-7,+r(7)[5]/46,sl,del(3)(q22)[13]/46,XY[2]	CMML→AML	
50	46,XY,del(7)(q11.1),t(9;22)(q34;q11.2)[10]/46,sl,-del(7),+r[9]/46,XY[1]	CML	ABL1 g.133738358 A>T (p.Y253F)<br>ABL1 g.133738363 G>A (p.E255K)<br>CHEK2 g.29130389 A>T (c.319+2T>A)
51	49,XX,t(3;9;22)(q27;q34;q11.2),+8,t(9;21)(q34;q21),+17,-19,+21,+r[20]	CML→AML	STAG2 g.123171416 C>T (p.R110*)<br>NSD1 g.176637865 C>G (p.S822C)<br>KMT2D g.49438042 G>A (p.T1710M)
52	49-50,XX,t(9;22;?)(q34;q11.2;?),+8,+17,-19,+der(22)t(9;22),+r[cp3]/46,XX[17]	CML→AML	
53	44~46,XY,add(5)(q?22),-9,del(11)(q13q23),del(20)(q11.2q13.2),dic(21;22)(p11.2;p11.2)del(21)(q22),+r[cp9]/ 46,XY[11]	CMML→AML	
54	46,XY,-18,+r[12]/46,XY[7]	MDS	IDH1 g.209113113 G>A (p.R132C)<br>ZRSR2 g.15827438 A>G (p.D185G)<br>TET2 g.106164907 A>C (p.T1259P)

			ZRSR2 g.1584124859 G>GGAGCCGGAGCCG (p.S445_R448dup)
55	45,XY,del(7)(q11.2),-11,-18,add(22)(p11.2),+r[7]	MDS	KMT2D g.49447416 G>C (p.R228G)<br>TP53 g.7577143 CAGT>C (p.L265del)
56	46,XY,t(1;21)(p32;q22),add(5)(q12),-7,del(19)(q13.2),+r[20]	MDS	
57	45,XY,der(3)r(3;?)(p26q29;?),der(5)t(5;21)(q14;q11.2),-21[20]	MDS	KIT g.55593628 G>T (p.G565V), 17p-^, 5q-^&
58	43,XY,-5,-7,-12,-17,add(19)(p13.1),-22,+r,+mar[10]/46,XY[10]	MDS	TP53 g.7577114 C>T (p.C275Y)
59	45,XY,del(5)(q13q33),-8[1]/46,sl,+mar[3]/47,sdl,+r[7]/46,XY[9]	MDS	ABL1 g.133760430 C>T (p.P918L)<br>CEBPA g.33792754 G>GGGC (p.P189dup)<br>TP53 g.7578413 C>T (p.V173M)<br>TP53 g.7579312 C>T (p.T125= (c.375G>A))
60	48,XY,-1,del(11)(q13q23),add(22)(q11.2),+r,+2mar[14]/46,XY[1]	MDS	
61	45,XY,der(5;13)(p10;q10),-7,der(11)t(7;11)(p13;q25),+r[14]/46,XY[6]	MDS	TP53 g.7577568 C>G (p.C238S)<br>ATM g.108224493 G>A (p.G2891D)
62	46~49,XY,-1,del(11)(q13?q23),add(22)(q12),+1~2r,+1~3mar[19]/46,XY[1]	MDS	
63	44,XY,-3,del(5)(q15q33),-7[10]/45,sl,add(6)(q13),+r[5]/47,sl,+8,+13,+22[5]	MDS	
64	43,XY,-5,-7,-12,-17,add(19)(p13.1),-22,+r,+mar[1]/42,idem,-Y[6]/46,XY[13]	MDS	
65	47~49,XY,-1,del(11)(q14),+del(11)(q14),add(22)(q11.2),+1~3r,+1~2mar[cp20]	MDS	CBL g.119149251 G>C (p.R420P)<br>SETBP1 g.42531907 G>A (p.D868N)
66	46,XY,del(1)(p31p13),t(1;12)(q31;p12),del(5)(q13q33)[13]/46,sl,r(2)[2]/46,XY[5]	MDS	JAK2 g.5073770 G>T (p.V617F)
67	43~45,XY,-3,add(5)(q12),del(5)(q13q33),+i(5)(p10),?r(6)(p23q21),-7,+22[cp18]/46,XY[2]	MDS	TP53 g.7577539 G>A (p.R248W)<br>TP53 g.7579575 G>A (p.Q38*)
68	47~49,XY,der(1)add(1)(p13)add(1)(q42.1),del(11)(q13q23),add(22)(q11.2),+r,+1~2mar[20]	MDS	
69	44,XY,add(3)(p13),der(4)t(4;11)(p15;q13),add(5)(q11.2),-7,-11,-17,+r[6]/45,sl,+mar[3]/46,XY[8]	MDS	
70	46,XX,+1,der(1;22)(q10;q10)[8]/46,XX,+1,der(1;13)(q10;q10)[2]/46,XX,r(7)(p?22q?22)[2]/46,XX[7]	MDS	SF3B1 g.198266834 T>C (p.K700E)<br>TET2 g.106197207 G>A (p.W1847*)<br>TET2 g.106164785 GC>G (p.H1219fs)<br>TET2 g.106197309 A>T (p.H1881L)
71	43~45,XY,-5,der(7)?inv(7)(p13;q11.2)t(7;15)(q21;q13),psu idic(12)(p13),-13,-15,-18,+1~3mar,+r[20]	MDS	
72	47~48,XY,-1,del(11)(q13q23),add(22)(q11.2),+1~2r,+mar[13]/48~49,sl,+del(11)(q13q23)[4]/46,XY[3]	MDS	
73	45-46,XY,t(1;21)(p32;q22),add(5)(q12),-7,add(19)(p13.3),del(19)(q13.2),+r,+1~2dmin[cp8]/46,XY[12]	MDS	
74	44,XY,add(1)(p36.3),-3,der(5)t(3;5)(q11.2;q14),-7,t(9;17)(p21;p13),del(13)(q12q14),-15,+r[19]/46,XY[1]	MDS	
75	47~48,XY,der(1)add(1)(p13)add(1)(q42.1),del(7)(p13p15),del(11)(q13q23),add(22)(q11.2),+2r,+mar[cp20]	MDS	
76	44~46,X,-Y,-5,-7,add(12)(p11.2),i(17)(q10),add(18)(q23),add(19)(p13.1),add(21)(p11.2),+r,+1~2mar[cp20]	MDS	ASXL1 g.31021211 C>T (p.R404*)<br>TP53 g.7578442 T>C (p.Y163C)
77	45~50,XY,add(2)(q33),-3,-5,-7,der(7)add(7)(p13)add(7)(q22),del(11)(p15.1p15.5),-18,-20,+r,+2~8mar[cp20]	MDS	
78	46,XY,t(5;7)(p13.3;p13),del(20)(q13.1)[1]/47,sl,del(9)(q13),-21,+r,+mar[4]/47,sdl,del(3)(q21q25)[6]/46,XY[8]	MDS	TP53 g.7577058 C>A (p.E294*)<br>TP53 g.37578190 T>C (p.Y220C)
79	45,XY,-3,-7,add(9)(q22),-12,-18,del(20)(q13.1),+r,+mar1,+mar2[5]/47,sl,+8,+18,+19,-r,-mar2,+mar3[13]/<br>46,XY[2]	MDS	TP53 g.7578211 C>A (p.R213L)
80	43~44,X,-Y,add(3)(p13),-5,-7,add(7)(q11.2),del(12)(p11.2p12),-13,-18,dic(20;22)(q11.2;p11.2),+2~3r,+mar[19]/<br>46, XY[1]	MDS	
81	44~54,XX,+1,-1,+4,-5,der(5)t(5;8)(p15.3;q13),+6,+8,-8,+9,-10,+11,add(11)(q13),-18,+19,idic(22)(p11.2),+1~2r,<br>+mar1, +mar2,+mar3[cp20]	MDS	TP53 g.7578190 T>C (p.Y220C)<br>TP53 g.7579329 T>C (p.K120E)

			ATM g.108165669 C>A (p.L1598I)
82	41~44,XX,del(1)(p35p36.2),del(3)(p21),-4,-5,der(5)ins(5;?)(p13;?),-7,add(12)(p11.2),der(12;15)(q10;q10),-15, -18, +1~2r,+1~2mar[cp17]/46,XX[3]	MDS	TP53 g.7578479 G>GT (p.P151Tfs)<br>TP53 g.7577539 G>C (p.R248G)
83	47,XY,t(1;6)(p36.1;q25),add(9)(p21),+11,del(16)(p13.1)[9]/46,XY,t(1;21)(p32;q22),add(5)(q12),-7, del(19)(q13.2),+r[5]/47,XY,+?del(11)(q13)[3]/46,XY[3]	MDS	
84	44,XX,del(4)(q21.3q31),add(5)(q11.2),add(6)(p23),-7,i(11)(q10),add(13)(p10),-14,-17,-18,add(18)(q21), del(20) (q13.1q13.3),del(21)(q22),+r[10]/46,XX[10]	MDS	
85	44~45,XY,-5,-7,add(12)(p11.2),i(17)(q10),+r,+mar[cp10]/44,XY,i(1)(p10),add(4)(q12),-5,-7,-10,add(11)(q25), add(15)(p11.2),-19,add(21)(p11.2),-21,-22,+4mar[2]/46,XY[9]	MDS	
86	44~49,XY,add(2)(q33),del(3)(q13.1),-5,der(7)add(7)(p13)add(7)(q22),der(7)del(7)(p12)del(7)(q32q36), del(11) (p15.1p15.5), t(12;18)(q23;p11.3),-18,-20,+r,+1~4mar[cp19]/46,XY[1]	MDS	TP53 g.7577571 A>T (p.M237K)<br>TP53 g.7578371 C>T (p.G187S)<br>ZRSR2 g.15841230 C>CAGCCGG (p.S447_R448dup)
87	45~46,XX,del(5)(q15),-7,add(8)(q24),add(11)(q23),der(11)t(11;15)(q25;q11.2),der(12)t(12;13)(p11.2;q12) add(13)(q34),t(12;13)(p11.2;q12),add(15)(p11.2),del(15)(q11.2q15),+r,+1~2mar[cp20]	MDS	TP53 g.7577123 (p.E271V)<br>TP53 g.7578523 (p.Q136P)
88	39~45,XX,-1,add(3)(p13),add(4)(q21),del(5)(q15q33),add(7)(q11.2),-10,add(12)(q21),-16,-17,-19, add(19)(p13.3),add(21)(q22),+r,+1~2mar[cp10]/71~86<4n>,slx2,-add(12),add(15)(p11.2),-22,-r[cp4]/46,XX[6]	MDS	TP53 g.7577120 C>T (p.R273H)<br>NF1g.29553477 A>AC (p.I679fs)
89	43~45,XY,add(1)(p36.1),add(5)(q11.2),dic(6;20)(p23;q13.3),idic(8)(p23),add(9)(q22),del(11)(q23),der(11) add(11)(p15)add(11)(q23),-13,-14,-16,der(16)t(16;17)(p13.1;q11.2),-17,-18,add(22)(p11.1),+r,+2~4mar[20]	MDS	TP53 g.7577145 G>GTAGA (p.L265Sfs)<br>TP53 g.7578455 C>G (p.A159P)
90	45,XY,t(2;3)(p13;p25),add(4)(q12),del(5)(q15q33),der(11)add(11)(p15)add(11)(q23),der(15;17)(q10;q10)[4]/ 44,XY,-2,t(2;11)(q12;p15),del(5)(q15q33),add(11)(q14),der(13;18)(q10;q10), add(14)(p11.2), der(15;17)(q10;q10),+r[5]/ 44,XY,-1,der(2)t(2;11)(q12;p15),del(5)(q15q33), add(11)(q13), der(11)t(2;11)(q12;p15)t(1;2)(p13;q33),der(14)t(1;14)(q12;p11.2)ins(14;?)(p11.2;?)[11]	MDS	TP53 g.7579414 C>T (p.W91*)<br>ETV6 g.12006381 C>T (p.L117F)
	**Lymphoid-Derived**		
91	45,XX,add(3)(q12),-7,-9,t(9;22)(q34;q11.2),-12,+der(?)t(?;3)(?;q13),+r[18]/46,XX[2]	ALL	
92	43~47,XY,der(3)t(3;4)(q?21;q?21),-4,+8,add(8)(p21)x2,-13,?14,+15,-17,del(17)(p?13),+r[cp11]	CLL	
93	45,XY,add(1)(q43),dup(1)(q12q42),-4,add(8)(p11.2)x2,+del(8)(q24),del(12)(p11.2p12),-13,-22,+r[5]/46,XY[15]	MM	
94	47,XY,+i(X)(p10),der(9)t(9;11)(p21;q23)del(9)(p13),der(11)t(9;11)(p21;q23),psu dic(15;15)(p11.2;q26.3),+17, der(17)t(8;17)(q21.2;p11.2)x2,-18,+r[16]/46,XY[4]	B-ALL	TP53 g.7577095 G>C (p.D281E)
95	41,X,-Y,add(1)(p13),der(3;13)(q10;q10),-4,der(6)t(6;15)(q21;q14),add(8)(p11.2),-10,del(13)(q12q22), add(16) (q11.2),-22,-22,+r[2]/41,sl,+add(1)(p12),-del(13)[15]/46,XY[3]	MM	
96	41~44,XY,+1,add(1)(q12),del(1)(p22p12),dic(1;14)(p11;p11.2),del(2)(p11.2),dic(7;12)(p11.2;p11.2),-8, del(12)(p13p11.2),psu dic(12;7)(q24.3;p15),-13,-14,+r[cp13]/46,XY[7]	MM	
97	43,X,-Y,add(1)(q12),der(1)add(1)(p34)add(1)(q21),inv(2)(q31q35),der(6)t(1;6)(q21;q22), der(8)t(1;8)(q21;q24.3), del(8)(p22),-13,-16,-20,-22,+r,+mar[17]/46,XY,inv(2)(q31q35)[3]	MM	1q+^, IGH rearrangement[&]
98	42~44,X,-Y,add(2)(q33),+3,add(3)(p25),del(3)(p14p21),+4,-6,dup(7)(q?11.1q36),-8,add(8)(q24.3), add(9)(q22)x2,der(9)add(9)(p24)add(9)(q22),add(12)(p13),-13,add(14)(p11.2),add(14)(q32),-15, add(17)(p11.2),-20,-22,-22,+r, +1~2mar[cp10]/46,XY[10]	MM	

Acute lymphoblastic leukemia (ALL), acute myeloid leukemia (AML), chronic myeloid leukemia (CML), chronic myelomonocytic leukemia (CCML), myelodysplastic syndrome (MDS), and multiple myeloma (MM). ^ by SNP microarray; [&]by fluorescence *in situ* hybridization.

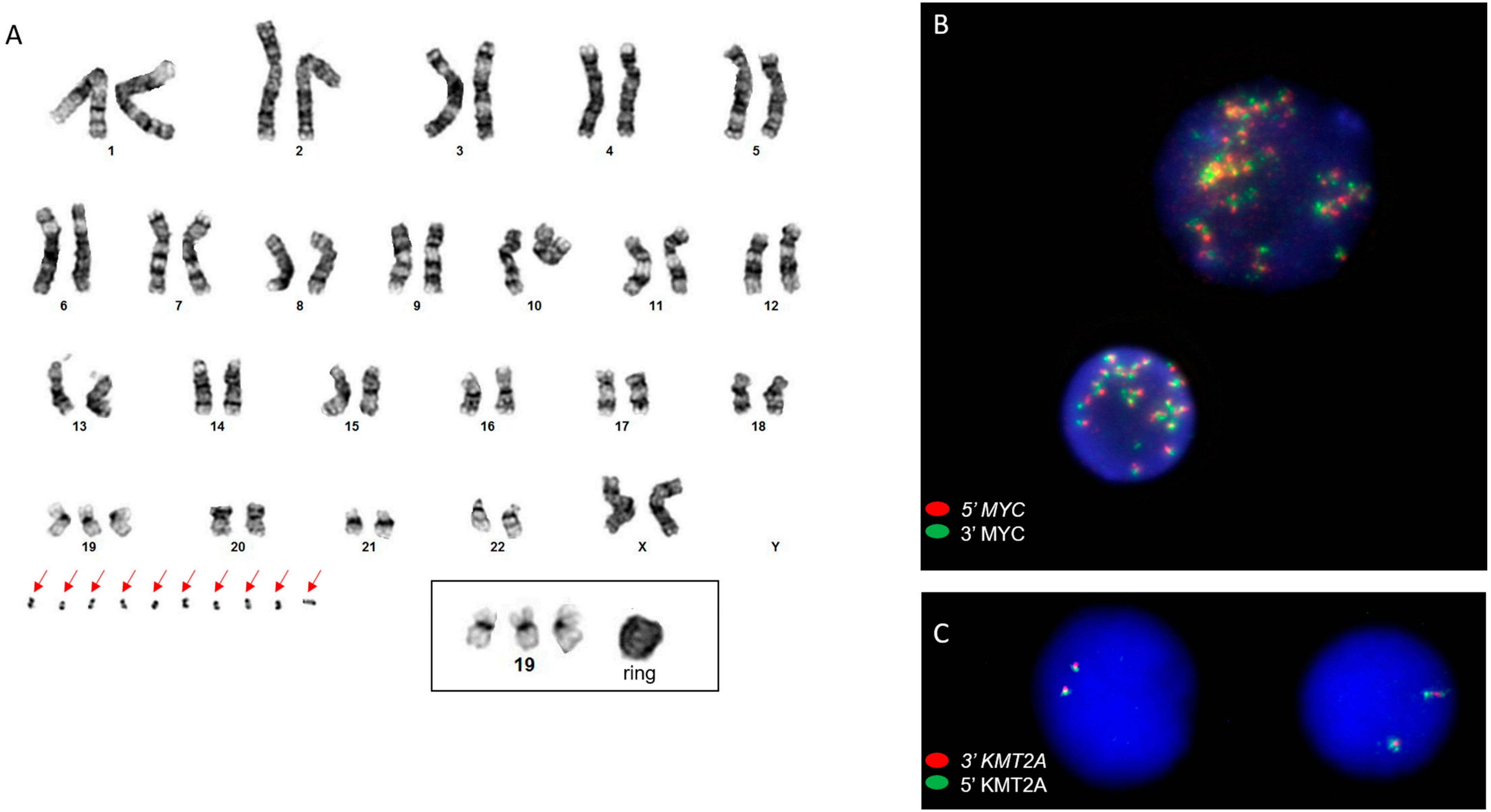

Figure S1. Double minute chromosomes in RC patients. (**A**) Karyogram with red arrows indicating double minutes. The inserted box of a partial karyogram shows trisomy 19 and gain of a ring chromosome of unknown origin (case ID 21). (**B**) FISH using a MYC break-apart probe-set revealed *MYC* amplification (case ID 21). (**C**) FISH using a *KMT2A* break-apart probe-set revealed a normal (two) copy number of *KMT2A* (case ID 73).

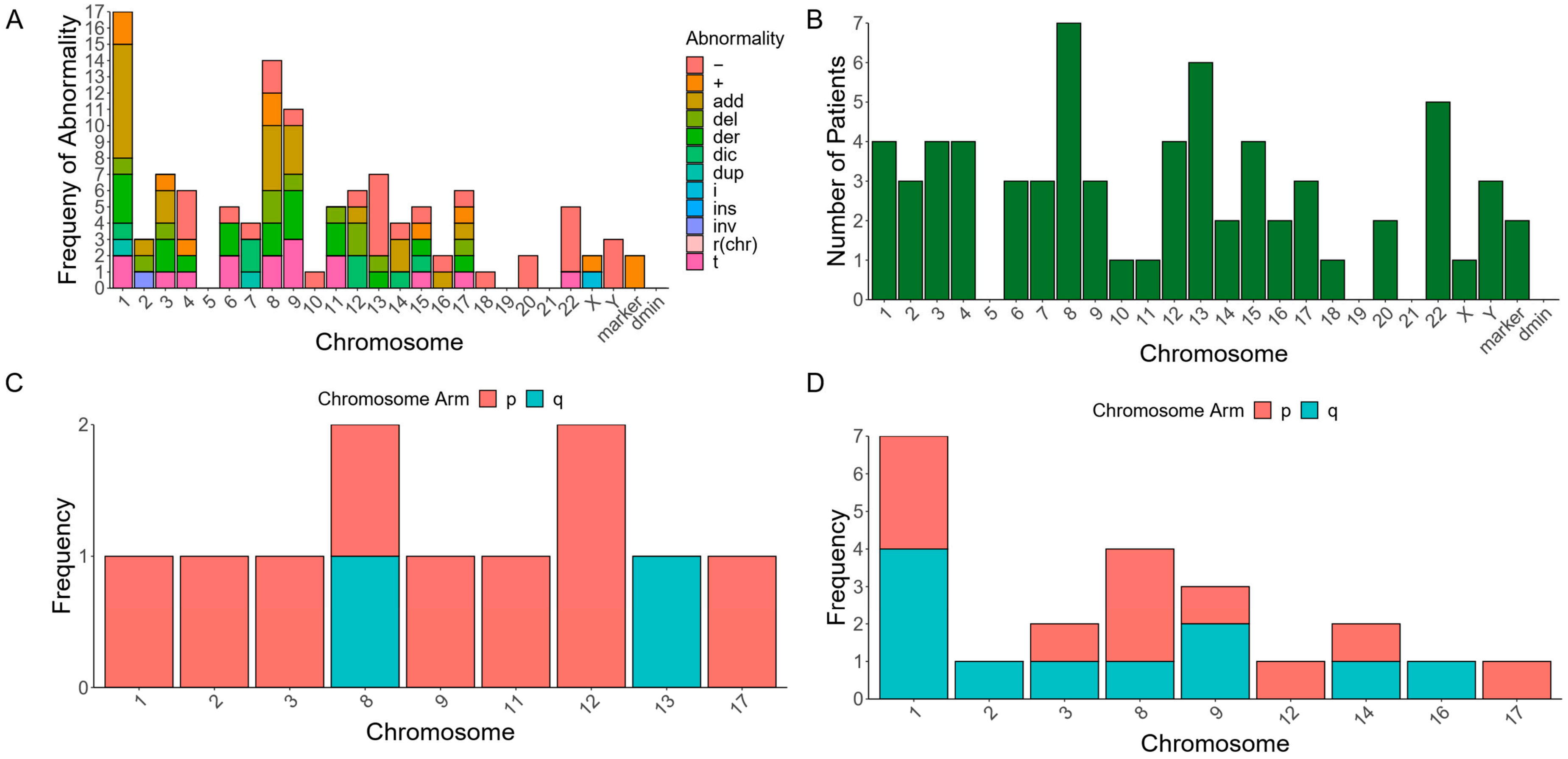

Figure S2. (A) A distribution of the frequency that specific chromosomal abnormalities are seen among 8 karyotypes from patients with lymphoid-derived cancers and RC (dmin = double minute chromosome; - = chromosomal deletion; + = chromosomal addition; add = addition of unknown origin; del = segmental deletion; der = derivative chromosome; dic = dicentric chromosome; dup = segmental duplication; i = isochromosome; idic = isodicentric chromosome; ins = insertion; inv = inversion; r(chr) = chromosome-derived RC; t = translocation). (**B**) A distribution of the number of lymphoid-derived cancer patient karyotypes with at least one structural abnormality for a given chromosome. (**C**) The frequency of segmental deletions and (**D**) unknown additions seen in the p arms and q arms of affected chromosomes, across 8 karyotypes from patients with lymphoid-derived cancers.

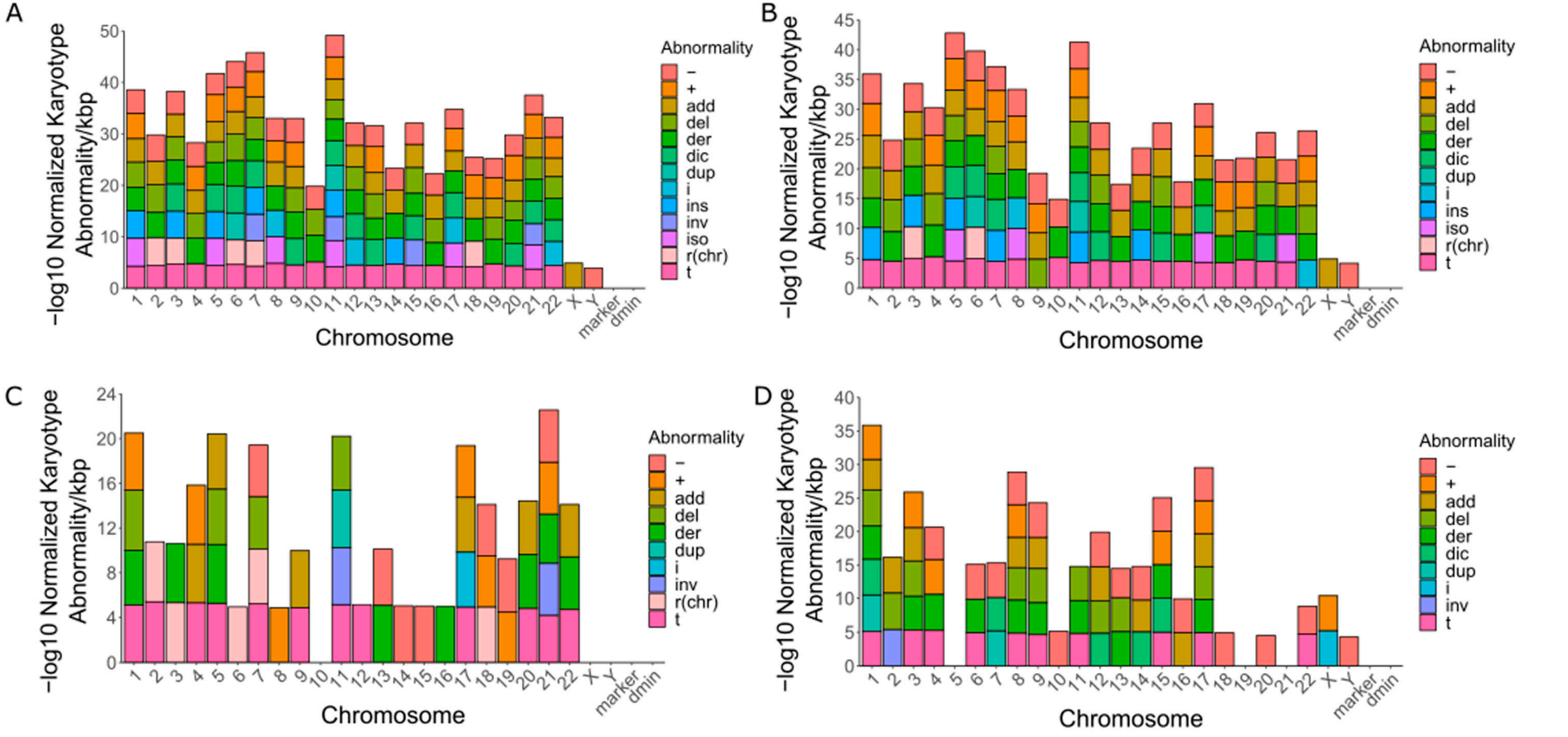

Figure S3. Chromosome size-normalized distribution of the frequency that specific chromosomal abnormalities are seen among (**A**) 90 karyotypes from patients with myeloid-derived cancers and RC; (**B**) 39 karyotypes from patients with *TP53* mutations, myeloid-derived cancers, and RC; (**C**)19 karyotypes from patients without *TP53* mutations, myeloid-derived cancers, and RC; (**D**) 8 karyotypes from patients with lymphoid-derived cancers and RC (dmin = double minute chromosome; - = chromosomal deletion; + = chromosomal addition; add = addition of unknown origin; del = segmental deletion; der = derivative chromosome; dic = dicentric chromosome; dup =

segmental duplication; i = isochromosome; idic = isodicentric chromosome; ins = insertion; inv = inversion; r(chr) = chromosome-derived RC; t = translocation).

Table S2. TP53 mutations and co-occurring structural abnormalities on chromosome 17, as depicted in Figure 4.

***TP53* Het / Compound Het Mutation; no Chr17 abnormality**	***TP53* Mutation(s) ; LOH -17**	***TP53* Mutation(s) ; LOH add(17)**	***TP53* Mutation(s) ; LOH der(17p)**	***TP53* Mutation(s); LOH i(17q)**	***TP53* Mutation ; Multiple Chr17 Structural Abnormalities**	***TP53* Mutation; LOH dic(17)**	**Chr17 LOH; No *TP53* Mutation**
p.M237K; p.G187S	c.994-1G>A; p.P58fs; -17	c.919+1G>C ; add(17)(p11.2)	p.H179Y; der(5;17)(p10;q10)	p.Y163C; i(17)(q10)	p.L145P; -17; add(17)(p11.2)	p.K164E; dic(5;17)(q11.2;p11.2)	idic(17)(p12)
p.R248W; p.Q38*	p.R273L; -17; SNP 17p CN-LOH	p.S127T; p.R248W; add(17)(p11.2)	p.W91*; der(15;17)(q10;q10)		p.C238Y; p.H193R; -17; add(17)(p13)		i(17)(q10)
p.E271V; p.Q136P	p.L265fs; p.A159P; -17	p.F270C; add(17)(q21)					SNP 17p-, 5q-
p.P151Tfs*; p.R248G	p.C275Y; -17	p.S215G; add(17)(p13)					
p.Y126C; p.H168R	p.L344P; -17						
p.Y220C; p.K120E	p.G245S; -17						
p.R248Q; p.A161T	p.R273H; -17						
P.E294*; p.Y220C	p.R273H; -17						
p.V173M; p.T125=(c.375G>A)	p.H193L; -17						
p.M237K; p.G187S	p.Y220C; -17						
p.R213L	p.H179R; SNP 17p-						
p.R248W							
p.G245S							
p.R248W							
p.Y205D							
p.C238S							
p.G334R							
p.H179R							
p.L265del							
